# Acoustic Characteristics of Voice and Speech in Post-COVID-19

**DOI:** 10.3390/healthcare13010063

**Published:** 2025-01-01

**Authors:** Larissa Cristina Berti, Marcelo Gauy, Luana Cristina Santos da Silva, Julia Vasquez Valenci Rios, Viviam Batista Morais, Tatiane Cristina de Almeida, Leisi Silva Sossolete, José Henrique de Moura Quirino, Carolina Fernanda Pentean Martins, Flaviane R. Fernandes-Svartman, Beatriz Raposo de Medeiros, Marcelo Queiroz, Murilo Gazzola, Marcelo Finger

**Affiliations:** 1 Departament of Speech, Language and Hearing Sciences, Faculty of Philosophy and Science, São Paulo State University (UNESP), Marília 17525-900, SP, Brazil; luana.cristina@unesp.br (L.C.S.d.S.); julia.vasquez@unesp.br (J.V.V.R.); v.morais@unesp.br (V.B.M.); tatiane.almeida@unesp.br (T.C.d.A.); leisi.sossolote@unesp.br (L.S.S.); jose.quirino@bp.org.br (J.H.d.M.Q.); carolina.pentean@unesp.br (C.F.P.M.); 2 Department of Computer Science, Institute of Mathematics and Statistics, University of São Paulo (USP), São Paulo 05508-220, SP, Brazil; marcelo.gauy@usp.br (M.G.); mqz@ime.usp.br (M.Q.); mfinger@ime.usp.br (M.F.); 3 Department of Classical and Vernacular Literature, Faculty of Philosophy, Language, Literature and Human Sciences, University of São Paulo (USP), São Paulo 05508-220, SP, Brazil; flavianesvartman@usp.br; 4 Department of Linguistics, Faculty of Philosophy, Language, Literature and Human Sciences University of São Paulo (USP), São Paulo 05508-220, SP, Brazil; biarm@usp.br; 5 Department of Computer Science, Faculty of Computing and Informatics, Mackenzie Presbyterian University (MACKENZIE), São Paulo 01302-907, SP, Brazil; murilo.gazzola@mackenzie.br

**Keywords:** post-acute COVID-19 syndrome, speech acoustics, voice, phonetics, adult

## Abstract

Background/Objectives: The aim of this paper was to compare voice and speech characteristics between post-COVID-19 and control subjects. The hypothesis was that acoustic parameters of voice and speech may differentiate subjects infected by COVID-19 from control subjects. Additionally, we expected to observe the persistence of symptoms in women. Methods: In total, 134 subjects participated in the study, were selected for convenience and divided into two groups: 70 control subjects and 64 post-COVID-19 subjects, with an average time of 8.7 months after infection. The recordings were made using the SPIRA software (v.1.0.) on cell phones, based on three verbal tasks: sustained production of the vowel/a/, reading a sentence, and producing a rhyme. Acoustic analyses of speech and voice were carried out with the PRAAT software (v.4.3.18), based on the following parameters: total sentence duration, number of pauses, pause duration, f_0_, f_0_SD, jitter, shimmer, and harmonics-to-noise ratio (HNR). Results: Regarding the acoustic characteristics of speech, there were no differences between the groups or between the sexes. Regarding the acoustic characteristics of voice, jitter, shimmer, and HNR, significant differences between the groups were found. Differences between sexes were observed in the following frequency-related parameters: f_0_, f_0_SD, and jitter. Conclusions: Some acoustic characteristics of the patients’ voice may show a deteriorated condition even after exacerbation of the disease. These characteristics are compatible with some of the symptoms reported by post-COVID-19 subjects, such as the presence of tension and fatigue. These voice acoustic parameters could be used as biomarkers to screen voice disorders in long-COVID, using artificial intelligence (AI), accelerating the search for diagnosis by specialists.

## 1. Introduction

During the COVID-19 pandemic, SPIRA project researchers developed methods to identify respiratory insufficiency using acoustic signals and artificial intelligence [1].

Researchers from this project also described which acoustic parameters, related to speech and voice fundamental frequency, distinguished COVID-19 patients from healthy individuals. A great number of pauses, longer pause duration, and higher values of f_0_ and f_0_SD characterized the speech and voices of patients compared to the controls [2,3]. These acoustic parameters are being tested as biomarkers to screen people with respiratory insufficiency.

However, there is a paucity of information on the acoustic characteristics of speech and voice that persist after recovery. Within the scope of SPIRA, the present study aims to compare voice and speech acoustic characteristics between subjects infected by COVID-19 who were not hospitalized (post-COVID-19 subjects) and control subjects.

It is well established in the literature that a large proportion of subjects affected by COVID-19 present persistent symptoms after the period of infection, characterizing the long-COVID or post-COVID-19 syndrome [4]. Post-COVID-19 syndrome is described as persistent symptoms in patients who have recovered from COVID-19, but who still have symptoms lasting longer than two weeks [5].

According to the WHO, “Post-COVID-19 occurs in individuals with a history of probable or confirmed SARS-CoV-2 infection, typically 3 months after onset, with symptoms lasting at least 2 months and unexplained by other diagnoses” [6]. Common symptoms include fatigue, shortness of breath, cognitive dysfunction, and others, generally impacting daily functioning [7,8].

According to a study [4], over 100 symptoms can occur after acute COVID-19, affecting cardiovascular, respiratory, musculoskeletal, and neurological systems. These symptoms have been noted in both hospitalized and non-hospitalized patients.

Studies have reported, with varying prevalence, the presence of non-specific symptoms such as fatigue, muscle pain, poor sleep, cough, shortness of breath, worsening quality of life, and impact on mental health [9,10,11]; as well as more specific symptoms, such as the presence of dysphonia, dysphagia, and hearing loss [10,12,13,14].

It is interesting to note that post-COVID-19 syndrome has been more commonly reported in women [5,7,8,9], with a 3-fold higher risk of being diagnosed with post-COVID-19 [15]. Some studies [15,16,17] have shed light on the issue of prevalence in women. Explanations take into account the exacerbated response of women’s immune systems, associated with hormonal influences. Women have higher levels of inflammatory cytokines compared to men. In addition, both hormone and stronger IgG antibody production in females may play a role in perpetuating the hyperinflammatory status of the acute phase even after recovery.

Regarding the characterization of more specific symptoms, researchers [7,10] reported that 41.7% of patients admitted to the Intensive Care Unit (ICU) presented symptoms related to communication difficulties, voice changes, swallowing difficulties, and laryngeal sensitivity, including persistent cough.

A posterior study [14] observed that 87.17% (68/78) of patients reported, after 15 days of post-COVID-19 recovery, symptoms related to speech, swallowing, and hearing problems. The most prevalent complaints related to voice and speech (present in 17.64% of patients) were changes in voice quality and intensity, shortness of breath when speaking, tension in the neck muscle when speaking, in addition to vocal fatigue. In a recent study, researchers found that most patients (25 out of 30) used excessive force to produce voice, and a significant weakening of the ability to produce voice immediately after the disease in all subjects. In addition, 17 subjects broke phrases in speech production [18].

It is important to highlight that the COVID-19 virus affects not only the respiratory system, but may also attack internal organs, including the brain, causing neurological and/or psychiatric problems [19]. Thus, consequences of both respiratory infection and changes in brain function can lead to changes in voice and speech characteristics.

Respiratory tract infections affect the same systems and structures that are used for speech and voice production. The epithelium of the vocal folds has an important role in vocal health and respiratory epithelial cells are also the primary sites of respiratory virus replication [20]. So, respiratory infections lead to increased production of proinflammatory cytokines in the respiratory epithelium and the recruitment and activation of inflammatory cells [18], causing edema of the mucosa lining the vocal tract.

In addition, the so-called cytokine storm, which may be the result of an extremely strong immune system response, attacks not only pathogenic cells, but also healthy ones. In addition, an immune attack on synaptic connections in the brain can lead to changes in its functioning. Patients complain of problems with memory, association-making, and concentration [19].

It is noted, however, that most studies that sought to describe post-COVID-19 symptoms were carried out on patients who had been hospitalized [7,10,11,12,13]. Authors of one study [8] were the first to highlight the unmet health needs of a large group of non-hospitalized patients after COVID-19 infection. Specifically, the authors reported an average of six persistent symptoms per patient, of which fatigue and dyspnea were also the most common among non-hospitalized patients.

Some authors [7,21] note the lack of systematic research on non-hospitalized COVID-19 patients and warn that post-COVID-19 syndrome may be more common in those with milder acute illness.

Therefore, it is essential to further explore the persistence of symptoms in non-hospitalized patients, especially those related to speech production, focusing on voice and speech aspects.

Considering that the main persistent symptoms related to speech and voice are characterized by changes in vocal quality, tension, vocal fatigue, and shortness of breath when speaking, and additionally, that post-COVID-19 syndrome has been more commonly reported in women, it is hypothesized that acoustic parameters of voice and speech differentiate subjects infected by COVID-19 from those control subjects (H1); and the persistence of symptoms in women should be observed (H2).

Based on this study, it is assumed that the results obtained in this investigation could provide important data to be used as biomarkers by AI in screening subjects with persistent symptoms, accelerating the search for diagnosis by specialists.

## 2. Materials and Methods

### 2.1. Subject

In total, 134 subjects participated in this study, were selected for convenience, and divided into two groups: 70 control subjects and 64 subjects who had been affected by COVID-19 (post-COVID-19 subjects).

The subjects were selected in the region where the authors lived, that is, in several regions of the State of São Paulo (Marília, Paulínea, São Paulo, Potim and Aparecida do Norte) and were recorded over 3 months, from April to July 2022.

To select the subjects, an initial interview was carried out, with questions directed at the subjects themselves, to check the established inclusion and exclusion criteria.

The inclusion criteria adopted for the selection of post-COVID-19 subjects were as follows: (1) being over 18 years old; (2) having had COVID-19 infection, confirmed by RT-PCR tests (reverse transcription followed by polymerase chain reaction), Rapid Antigen Test (TR-Ag) or Antigen Self-Test (AT-Ag); (3) having presented mild to moderate symptoms; (4) having not required hospitalization during the period of infection. We considered as mild to moderate symptoms the presence of some characteristics: respiratory symptoms (runny nose, sore throat, cough), fever, headache, loss of taste/smell, fatigue and muscle aches, and/or diarrhea, with normal oxygen saturation (>92%) and no need for hospitalization.

Those subjects who (1) did not remember the date on which they had COVID-19 infection; (2) who had voice and/or speech complaints before COVID-19 infection; and (3) who presented some alteration (neurological, cognitive, psychological, etc.) that prevented them from understanding the questions in the initial interview and/or from carrying out the tasks requested at the time of collection were excluded from the sample. The average post-COVID-19 time was 8.7 months (±6.27).

The inclusion criteria for selecting control subjects were as follows: (1) being over 18 years old, (2) not having presented any symptoms of COVID-19 infection, proven through tests; (3) not reporting or presenting any change in communication, especially involving voice and speech. Additionally, the control subjects were selected so that they could be matched with the post-COVID-19 subjects in terms of sex and age group.

Although the initial projection was the participation of 100 control subjects and 100 post-COVID-19 subjects, many of the recruited subjects did not meet the established criteria and, therefore, could not be included in the sample.

Table 1, below, presents the final distribution of participating subjects, according to age group and sex.

### 2.2. Procedure

The same methodological procedures used in the Spira Project to collect the audio were carried out.

The recordings were made by the study’s co-authors linked to the area of Speech Therapy, using their respective cell phones, using the SPIRA software (v.1.0). The recordings were not made in acoustically treated rooms; however, priority was given to recording in silent environments. The cell phones were placed at 20 cm from the participant’s mouth.

Initially, the free and informed consent form was read to the subjects and asked if they would like to participate in the research. The subjects expressed their agreement verbally with the recording of the word “accepted”.

Subsequently, three verbal tasks were asked to be performed: the sustained production of the vowel [a], the reading of the phrase “o amor ao próximo ajuda a enfrentar essa fase com a força que a gente precisa” (love for others helps to face this phase with the strength that we need) and the production of a rhyme and/or an excerpt from a prayer.

In this study, only the first two tasks were considered in the analysis: the sustained production of [a] and reading the sentence.

To record the sustained vowel, the subject was asked to take a deep breath and then produce a very long [a] using their usual voice until they were out of breath (e.g., inhale and produce [aaaaaaaaaaaaaaaaaa]).

To read the sentence, the subjects were presented with the sentence displayed on the cell phone screen, positioned horizontally, always in the same format:

“O amor ao próximo ajuda a enfrentar (14 syllables) esta fase com a força que a gente precisa” (15 syllables).

The sentence was designed in such a way that, if pauses are made, they would occur according to prosodic constituency restrictions, that is, in two places where there is a big boundary between constituents, predicted by the grammar (after “next”: the subject of the clause; after “phase”: adjunct adverbial).

Through the Spira software (v.1.0), the files are recorded in WAV format and the audio signals were sampled at 48 kHz with 16-bit samples.

After the recordings, all files were acoustically inspected by five coauthors, using PRAAT software (v. 4.3.18), based on oscillogram and spectrogram analyses.

It was found, however, that eleven files (*n* = 11) related to sentence production had to be discarded due to reading issues, where subjects read slowly and demonstrated syllabic reading (*n* = 3 control subjects); and problems with corrupted files (*n* = 7 of the control subjects and *n* = 01 of the post-COVID-19 subjects).

Therefore, the distribution of speech samples can be seen in Table 2 below:

### 2.3. Analysis

Acoustic analyses of speech and voice were carried out using the PRAAT software (v. 4.3.18), based on the oscillogram and spectrogram. Although acoustic measurements of both speech and voice involve physical parameters related to amplitude, frequency and time, based on our previous studies [2,3], we prioritized the temporal parameter to analyze speech and the frequency and amplitude parameters to extract information about the voice.

The analysis of speech samples, extracted from the production of the sentence, was carried out based on the following three parameters:

(1) Total sentence duration: it refers to the time, measured in seconds, used by participants to produce the sentence. Speech production depends on the integrity of a complex coordination of several subsystems: neurolinguistic, aerodynamic, phonatory, oro-nasal, and articulatory [22]. Therefore, any change in one of these subsystems will result in temporal changes in speech production.
(2) Number of silent pauses: it refers to a period of silence that may occur in the speech production process. The presence of pauses fundamentally fulfills a linguistic function of organizing the utterance, that is, there are places provided by the grammar for them to occur. The excessive presence of silent pauses may suggest, among others, the presence of respiratory problems. The criteria adopted to define the pause included the presence of silence or low energy in the acoustic signal lasting more than 200 milliseconds [23].
(3) Duration of silent pauses: it refers to the period, measured in seconds, used to interrupt the flow of speech. Since the presence of pauses is linguistically motivated, their duration must be sufficient to mark the organization of the utterances. An increased duration of pauses may indicate, among others, respiratory problems. When the silent pause occurred (1) between two vowels, the boundaries were inserted at the left vowel last pulse and at the right vowel first pulse; (2) before unvoiced plosives, the burst was observed for the boundary location (the plosive silence portion was necessarily included in the overlapping silent pauses); and (3) before a fricative sound, the boundary was inserted at the beginning of the visible frication noise in the spectrogram.

The acoustic characteristics of the voice were made based on the selection of a stationary period of 3 s from sustained production of [a], according to the following five acoustic parameters:

(1) f_0_: it is related to the number of cycles per second performed by the vocal folds and is expressed in Hertz (Hz). Changes in the mass, length, and tension of the vocal folds modify the fundamental frequency [24].
(2) Standard deviation of f_0_: it is related to the amount of variation in the vibration frequency of the vocal folds. Asymmetric changes in the vocal folds, whether in mass or tension, as well as tremor or inefficiency of laryngeal muscle control, lead to a deviation in vibration and, consequently, changes in dpf_0_ [24].
(3) jitter: it refers to a measure of short-term frequency disturbance, that is, it indicates the cycle-to-cycle variation in vocal fold vibration frequency. Vocal fold vibration shows a low level of tremor. Higher levels of tremor are observed in pathological voices. Higher jitter values may be related to increased vocal fold stiffness, changes in glottal closure, asymmetry, and irregularity of vocal fold vibration. Reference values for normal voices are up to 1.04% [24,25].
(4) Shimmer: It refers to a measure of short-term amplitude disturbance, that is, it indicates the cycle-to-cycle variation in the vocal fold oscillation concerning the wave amplitude. Variation in shimmer is mainly detected when there is a change in the mass of the vocal folds, such as edema, polyps, or carcinomas. Increased values of this measure are related to harshness, hoarseness, and breathiness. Reference values for normal voices are up to 3.81% [24,25].
(5) Harmonics-to-noise ratio (HNR): it represents the degree of periodicity in a sound signal. It is an estimate of the relationship between the harmonic energy of the voice signal and the noise energy in the signal. HNR values are usually higher in normal voices than in pathological voices, since normal voices are louder than pathological voices. Decreased HNR values can provide important information about vocal deterioration. Changes involving glottal closure appear to be reflected in noise measurements. HNR values below 7 dB are considered pathological [24].

The stationary period of 3 s from vowel production is used to capture stable, representative vocal samples for acoustic analysis and consensual practice in voice analysis [25]. This period allows a consistent and reliable measurement of vocal parameters by minimizing transitions and noise from the onset and offset of the vowel sound. The 3 s window provides enough data to analyze without causing vocal strain, ensuring both consistency across samples and reduced influence of noise or unintended vocal variation.

## 3. Results

### 3.1. Acoustic Analysis of Speech

Table 3 presents the results of the acoustic analysis of speech, considering the group of control and post-COVID-19 subjects.

A series of factorial ANOVAs was conducted to examine the effect of group and sex on speech parameters. An alpha value of less than 0.05 was adopted.

In the analysis of total sentence duration, there was no significant difference for the group (F (1,118) = 1.57, *p* = 0.21), for sex (F (1,118) = 0.02, *p* = 0.87), or the interaction between group × sex (F (1,118) = 0.36, *p* = 0.54).

Regarding the number of pauses, participants showed very similar performance, with no significant effect for the group (F (1,118) = 0.12, *p* = 0.72), sex (F (1,118) = 0.05, *p* = 0.82), or the interaction between group × sex (F (1,118) = 0.10, *p* = 0.74).

As for the average pause duration, there were also no significant differences between group (F (1,118) = 0.07, *p* = 0.79), sex (F (1,118) = 1.30, *p* = 0.25), or the interaction between group × sex (F (1,118) = 0.47, *p* = 0.49).

In summary, there were no differences between the groups or sexes in terms of speech production. Participants behaved similarly when performing the linguistic task assigned to them, both in terms of sentence duration and the number and duration of pauses.

### 3.2. Acoustic Analysis of Voice

Table 4 below shows the results of the acoustic voice analysis, considering the control and post-COVID-19 subject groups.

Factorial ANOVAs were used to compare voice parameter measurements, considering the effects of group and sex. An alpha value of less than 0.05 was adopted.

Regarding fundamental frequency (f_0_), there was no significant difference between the groups (F (1,130) = 0.23, *p* = 0.63), nor for the interaction between group and sex (F (1,130) = 1.22, *p* = 0.27).

Regarding the standard deviation of fundamental frequency (dpf_0_), a significant effect was found only for sex (F (1,130) = 6.83, *p* < 0.01, partial η^2^ = 0.05), with women showing higher values than men. No significant differences were observed for the group (F (1,130) = 0.001, *p* = 0.97) or the interaction between group × sex (F (1,130) = 0.08, *p* = 0.76).

**Figure 1:**
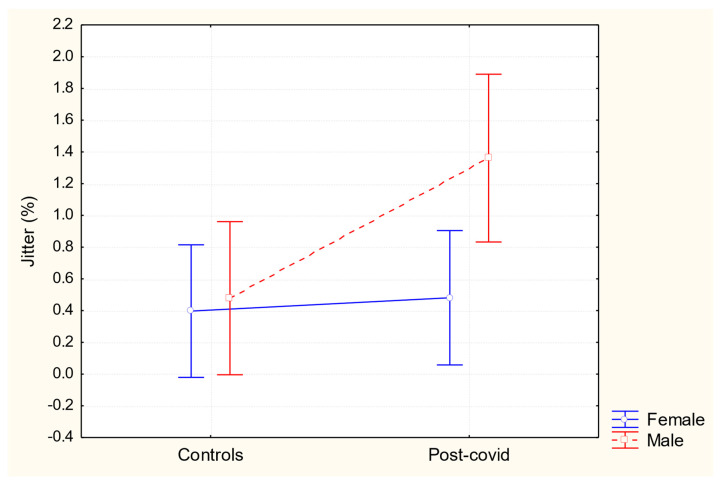
Jitter values according to the sex and group of participants.

**Figure 2:**
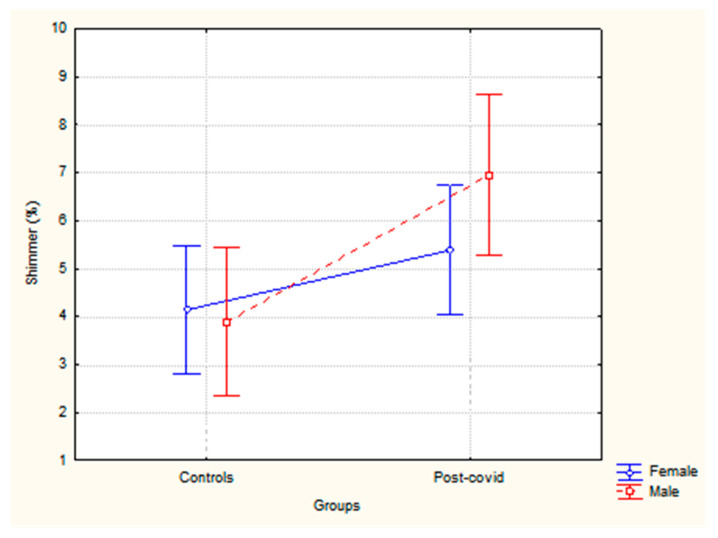
Shimmer values according to the sex and group of participants.

Regarding jitter, a significant effect was found for both group (F (1,130) = 4.22, *p* = 0.04, partial η^2^ = 0.03) and sex (F (1,130) = 4.17, *p* = 0.04, partial η^2^ = 0.03), but not for the interaction between group × sex (F (1,130) = 2.88, *p* = 0.09). Specifically, the post-COVID-19 group showed higher jitter values than the control group. Similarly, men had higher jitter values compared to women. Figure 1 below illustrates the results:

Regarding shimmer, a significant difference was found only for the group (F (1,130) = 8.26, *p* < 0.01, partial η^2^ = 0.06), with higher values for post-COVID-19 subjects, as illustrated in Figure 2. No differences were observed between sex (F (1,130) = 0.79, *p* = 0.37), nor for the interaction between group × sex (F (1,130) = 1.47, *p* = 0.22).

**Figure 3:**
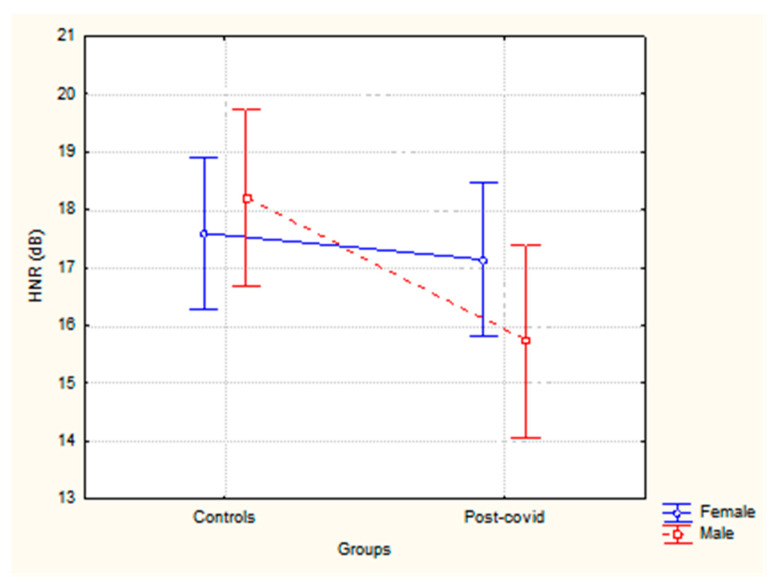
Harmonics-to-noise ratio (HNR) values according to the sex and group of participants.

In the analysis of the harmonics-to-noise ratio (HNR), a significant effect was found only for the group (F (1,130) = 3.92, *p* = 0.04, partial η^2^ = 0.03), with lower values for post-COVID-19 subjects, as illustrated in Figure 3. No differences were observed between sex (F (1,130) = 0.29, *p* = 0.59), nor for the interaction between group × sex (F (1,130) = 1.86, *p* = 0.17).

In summary, significant differences between the groups were observed in parameters related to cycle-to-cycle variation in frequency and amplitude (jitter and shimmer, respectively), as well as in the harmonics-to-noise ratio. Differences between sex were noted in the following frequency-related parameters: f_0_, f_0_ standard deviation, and jitter.

## 4. Discussion

According to the literature, a significant number of individuals who had COVID-19, particularly women, experience persistent symptoms after recovery [5,7,8,21]. These include both nonspecific symptoms, such as fatigue, muscle pain, poor sleep, cough, shortness of breath, reduced quality of life, and mental health impact [5,7,8,9,10,11], as well as more specific symptoms like dysphonia, dysphagia, and hearing loss [7,10,11,12,13].

Considering that the most frequent symptoms in non-hospitalized post-COVID-19 individuals are related to fatigue and dyspnea [8], and that the prevalence of these symptoms is higher in women [5,7,8,21], the hypotheses in this study were as follows: H1: some acoustic parameters of voice and speech could differentiate post-COVID-19 subjects from control subjects; H2: there would be a sex effect in the analysis of the investigated speech and voice parameters.

### 4.1. The Effect of the Group (Control vs. Post-COVID-19) on Voice and Speech Characteristics

The first hypothesis (H1), related to the difference between post-COVID-19 and control groups, was partially confirmed, since the voice parameters related to cycle-to-cycle variation in frequency and amplitude (jitter and shimmer, respectively), as well as the harmonics-to-noise ratio (HNR), distinguished the groups.

Regarding speech production, the analysis was based on the production of a relatively long sentence (29 syllables), where silent pauses could occur as conditioned by grammar. The results showed that none of the speech production parameters (total sentence duration, number of pauses, and pause duration) differentiated the groups. This contradicted the initial expectation that post-COVID-19 subjects might produce longer sentences with more pauses and longer pause durations than controls, due to the presence of fatigue and dyspnea.

These results partially contradict those reported in previous studies [14,25] addressing some sequelae in post-COVID-19 patients. In the first phase of that study [14], 87.17% (68/78) of patients reported symptoms related to speech, swallowing, and hearing issues 15 days after post-COVID-19 recovery, with the primary speech complaint (present in 17.64% of patients) being shortness of breath while speaking. However, in the second phase of the study, one month after the first phase (approximately 2.5 months post-COVID-19), 67.64% (46/68) of the subjects had recovered; and in the third phase (around 3.5 months post-COVID-19), 54.54% (12/22) of participants also recovered. However, 12 subjects (15.38% of the total participants) continued to have complaints related to speech, swallowing, and hearing. In another study [19], the authors reported that 17 subjects (17 out of 30, 56.6%) broke phrases in speech production. These subjects had developed communication disorders (voice, speech and/or language disorders) between 1 month and 12 months after recovery of COVID-19.

Considering that the average post-COVID-19 time for participants in the present study was 8.7 months (±6.27), it can be concluded that post-COVID-19 subjects were able to perform the linguistic task of producing a long sentence similarly to control subjects, without exhibiting any acoustic characteristics indicating difficulty in its production. However, it is important to note that this result cannot be generalized to other linguistic tasks that require greater respiratory and working-memory support, such as spontaneous conversation.

Regarding vocal parameters extracted from the sustained vowel [a], three of them—jitter, shimmer, and harmonics-to-noise ratio—showed differences between the post-COVID-19 and control groups. This supports complaints of changes in voice quality and intensity, neck muscle tension while speaking, and post-COVID-19 vocal fatigue.

The presence of vocal alterations in post-COVID-19 individuals has been described in several studies and summarized in a systematic review and meta-analysis [26], which highlights that 17.1% of COVID-19 patients experienced dysphonia after their recovery.

Jitter refers to the cycle-to-cycle variation in the frequency of vocal fold vibration. Higher jitter values can be related to increased vocal fold stiffness, changes in glottal closure, asymmetry, and irregular vocal fold vibration. The reference values for normal voices are up to 1.04%. It was observed that post-COVID-19 male subjects contributed to higher jitter values in their group compared to the control group, with an average of 1.36% above the expected range for normal voices. Although post-COVID-19 women showed higher jitter values than the control group women (0.48 and 0.39, respectively), these values are within the normal range and below those reported for Brazilian adults with normal voices [25].

Shimmer, on the other hand, refers to the cycle-to-cycle variation in the amplitude of vocal fold vibration. Shimmer variation is mainly detected when there is a change in vocal fold mass, such as edema, polyps, or carcinomas. Increased shimmer values are associated with roughness, hoarseness, and breathiness in the voice. The reference values for normal voices are up to 3.81% [25]. The results of the present study showed that the post-COVID-19 group had higher shimmer values compared to the control group and well above what is expected for normal voices (5.38% for women and 6.95% for men).

The harmonics-to-noise ratio (HNR) represents the degree of periodicity in a signal. It estimates the relationship between the energy of the harmonics in the voice signal and the energy of the noise in the signal. Decreased HNR values can provide important information about vocal deterioration. Changes involving glottal closure seem to be reflected in noise measurements. HNR values below 7 dB are considered pathological [24]. Although the results showed normal values for both groups (ranging from 15.72 to 18.20), the post-COVID-19 group had lower values compared to the control group. This can suggest that post-COVID-19 voices have more noise and less harmonic quality.

The few existing studies in the literature on vocal characteristics in post-COVID-19 individuals have reported contradictory results. While researchers in one study [27] found no differences in any acoustic voice measures between post-COVID-19 subjects (with an average post-COVID-19 time of 8.2 months) and control subjects, researchers in another study [28] reported increased jitter and shimmer values in post-COVID-19 subjects due to subacute inflammation of the glottis, also referred to as “long-lasting inflammation”.

As an explanatory hypothesis, the authors [28] suggest that “long-lasting inflammation” may result from the previous presence of SARS-CoV-2 in the larynx, including the vocal folds, due to the presence of the ACE-2 receptor, which is considered the SARS-CoV-2 receptor. Therefore, dysphonia as a COVID-19 symptom may occur due to the direct entry of SARS-CoV-2 into laryngeal cells, leading to inflammation of the larynx and vocal cords. In another study [29], researchers highlighted the unusual fact that acute laryngitis caused by COVID-19 lasts much longer than other viruses.

Other possible explanations for persistent dysphonia come from the study by Lechien et al. [30]. The authors suggest that other factors may be associated with vocal changes in post-COVID-19 individuals, such as laryngopharyngeal reflux or the use of inhaled corticosteroids. They also highlight that SARS-CoV-2 is known as a neurotropic virus and has been linked to some innervation disorders, such as paradoxical vocal fold movement, olfactory dysfunction, and sudden hearing loss. Therefore, some cases of dysphonia may be related to laryngeal nerve dysfunction rather than being solely attributed to persistent inflammation.

It is important to highlight that the size effects of post-COVID-19 on voice parameters showed small to medium impacts: 0.03 for jitter and HNR and 0.06 for shimmer. Partial eta squared (η^2^) values indicate the variability explained by group differences. Due to small effect sizes, factors like respiratory symptoms, psychological, neurological, muscular, and demographic aspects should also be considered in voice analysis.

### 4.2. The Effect of Sex (Male vs. Female) on Voice and Speech Characteristics

The second hypothesis (H2) was that the persistence of symptoms in women should be observed, since post-COVID-19 syndrome has been more commonly reported in women [5,7,8,9]. It seems reasonable to assume that trends observed in the acute phase of the disease may extend to the post-acute phase, although their effects may be reduced after recovery.

Our results did not confirm H2. Differences between sexes were observed in jitter in the opposite direction than expected.

The f_0_ values reported in the present study (mean of 187.46 Hz for women and 124.42 Hz for men, regardless of group) were expected and are compatible with those reported in a previous study [25] for Brazilian adults with normal voices.

Regarding the f_0_ standard deviation (dpf_0_), higher values were found for women compared to men. However, this difference was not specific to the post-COVID-19 group of women. Additionally, the values obtained for both control subjects (4.82 Hz for women and 1.40 Hz for men) and post-COVID-19 subjects (4.43 Hz for women and 1.71 Hz for men) are well below those reported for healthy subjects (8.87 Hz for women and 8.19 Hz for men) [24], suggesting minimal variation in vocal fold vibration.

Regarding jitter, the difference between the sexes was opposite to what was expected. Higher values were observed for men compared to women, with post-COVID-19 men showing a jitter value (1.36%) slightly above the normal range (up to 1.04%) [24,25]. Additionally, Spazzapan et al. [25] also reported jitter differences between sexes in normal voices. However, higher values were observed in women, explained by their fundamental frequency and vocal intensity.

Higher jitter values can be related to increased stiffness of the vocal folds, changes in glottal closure, asymmetry, and irregular vocal fold vibration [24,25]. An explanatory hypothesis for the increased jitter values in men may be linked to both their behavior related to social and cultural roles, as well as differences in their immune system [31,32].

The authors [31,32] explain that men are more likely to engage in health-damaging behaviors, such as smoking and alcohol consumption, and have higher rates of pre-existing comorbidities associated with poor COVID-19 outcomes, including hypertension, cardiovascular disease, and chronic obstructive pulmonary disease. Furthermore, the authors reported that a sex-stratified analysis showed that even after adjusting for age, the effect of comorbidities on COVID-19 mortality was greater for men than for women. The authors finally warn that although women have a generally stronger immune response, men are more prone to developing a cytokine storm associated with poor COVID-19 outcomes [32]. Again, we need to consider the small effect size (0.03) and should take into account factors like respiratory symptoms, psychological, neurological, muscular, and demographic aspects.

This study has several limitations. Firstly, there was no ideal balance between gender-related quantities. Additionally, although we used the Delphi consensus to determine the post-COVID-19 time, there was significant variability in this time among participants (the average post-COVID-19 time was 8.7 months with a standard deviation of ±6.27). Secondly, some technical limitations need to be considered. There was no uniformity in the type of cell phones used for data collection, nor strict control over the ambient noise where the data were collected. Efforts were made to collect the data in the quietest environment possible.

We also would like to highlight a limitation related to cross-sectional design. This type of design prevents the determination of causality. The effects observed between group and sex on some acoustic characteristics cannot be interpreted as a cause–effect relationship, as data were collected at a single point in time. Additionally, cross-sectional studies can be affected by selection bias and variability in seasonal or context factors, which may influence the results obtained and limit their generalization to other contexts or time periods.

## 5. Conclusions

Some acoustic characteristics of the voices of post-COVID-19 subjects may show different from normal values even after the exacerbation of the disease. These characteristics are consistent with some of the symptoms reported by post-COVID-19 subjects, such as tension and fatigue. The speech production characteristics do not distinguish post-COVID subjects from controls. Overall, there was no sex effect on the persistent voice characteristics of post-COVID-19 subjects, except for the jitter parameter.

These results can be used in screening systems to detect vocal deviations in post-COVID-19 subjects, thereby accelerating the diagnostic process by specialists.

## Figures and Tables

**Table 1:** Distribution of subjects according to sex and age group.

Ages	Control Subjects		Post-COVID-19 Subjects	
	Female	Male	Female	Male
≤40 years	10	10	12	12
41–50 years	10	06	12	03
51–60 years	10	07	09	05
≥60 years	10	07	06	05
Total	40	30	39	25

**Table 2:** Distribution of speech samples according to gender and age group.

Ages	Control Subjects		Post-COVID-19 Subjects	
	Female	Male	Female	Male
≤40 years	10	10	12	12
41–50 years	09	04	12	03
51–60 years	10	06	09	04
≥60 years	04	06	06	05
Total	33	26	39	24

**Table 3:** Descriptive analysis (mean and standard deviation) of speech acoustic parameters.

Speech Acoustic Parameters	Control Subjects		POST-COVID-19 Subjects	
	Female (*n* = 33)	Male (*n* = 26)	Female (*n* = 39)	Male (*n* = 24)
Total sentence length (seg)	5.94 (± 0.20)	6.11 (± 0.23)	5.80 (± 0.19)	5.70 (± 0.24)
Number of pauses	0.96 (± 0.18)	1.07 (± 0.20)	1.10 (± 0.17)	1.08 (± 0.21)
Duration of pauses (seg)	0.33 (± 0.04)	0.35 (± 0.04)	0.31 (± 0.03)	0.39 (± 0.04)

**Table 4:** Descriptive analysis (mean and standard deviation) of voice acoustic parameters.

Voice Acoustic Parameters	Control Subjects		Post-COVID-19 Subjects	
	Female (*n* = 40)	Male (*n* = 30)	Female (*n* = 39)	Male (*n* = 25)
f_0_ (Hz)	182.87 (± 5.20)	126.23 (± 6.01)	192.06 (± 5.27)	122.62 (± 6.58)
dpf_0_	4.82 (± 1.05)	1.40 (± 1.21)	4.43 (± 1.06)	1.71 (± 1.33)
Jitter (%)	0.39 (± 0.21)	0.47 (± 0.24)	0.48 (± 0.21)	1.36 (± 0.26)
Shimmer (%)	4.14 (± 0.67)	3.89 (± 0.77)	5.38 (± 0.68)	6.95 (± 0.85)
Harmonics-to-noise ratio (HNR) (dB)	17.59 (± 0.66)	18.20 (± 0.76)	17.13 (± 0.67)	15.72 (± 0.84)

## Data Availability

Data are available on request.
